# Acute health effects associated with satellite-determined cyanobacterial blooms in a drinking water source in Massachusetts

**DOI:** 10.1186/s12940-021-00755-6

**Published:** 2021-07-16

**Authors:** Jianyong Wu, Elizabeth D. Hilborn, Blake A. Schaeffer, Erin Urquhart, Megan M. Coffer, Cynthia J. Lin, Andrey I. Egorov

**Affiliations:** 1 Oak Ridge Institute for Science and Education participant at US EPA, Office of Research and Development, Research Triangle Park, Durham, NC 27711 USA; 2 US Environmental Protection Agency, Office of Research and Development, Research Triangle Park, Durham, NC 27711 USA; 3 Science Systems and Applications, Inc., NASA Goddard Space Flight Center, Greenbelt, MD USA; 4 Center for Geospatial Analytics, North Carolina State University, Raleigh, NC USA; 5 ICF International, Durham, NC 27713 USA

**Keywords:** Remote sensing, Drinking water, Human illness, Cyanobacteria, Harmful algal blooms, Satellite imagery, Respiratory, Gastrointestinal, Dermal

## Abstract

**Background:**

The occurrence of cyanobacterial blooms in freshwater presents a threat to human health. However, epidemiological studies on the association between cyanobacterial blooms in drinking water sources and human health outcomes are scarce. The objective of this study was to evaluate if cyanobacterial blooms were associated with increased emergency room visits for gastrointestinal (GI), respiratory and dermal illnesses.

**Methods:**

Satellite-derived cyanobacteria cell concentrations were estimated in the source of drinking water for the Greater Boston area, during 2008–2011. Daily counts of hospital emergency room visits for GI, respiratory and dermal illnesses among drinking water recipients were obtained from an administrative record database. A two-stage model was used to analyze time-series data for an association between cyanobacterial blooms and the occurrence of illnesses. At the first stage, predictive autoregressive generalized additive models for Poisson-distributed outcomes were fitted to daily illness count data and daily predictive variables. At the second stage, residuals from the first stage models were regressed against lagged categorized cyanobacteria concentration estimates.

**Results:**

The highest cyanobacteria concentration (above the 75th percentile) was associated with an additional 4.3 cases of respiratory illness (95% confidence interval: 0.7, 8.0, *p* = 0.02, *n* = 268) compared to cyanobacteria concentrations below the 50th percentile in a two-day lag. There were no significant associations between satellite derived cyanobacterial concentrations and lagged data on GI or dermal illnesses.

**Conclusion:**

The study demonstrated a significant positive association between satellite-derived cyanobacteria concentrations in source water and respiratory illness occurring 2 days later. Future studies will require direct measures of cyanotoxins and health effects associated with exposure to cyanobacteria-impacted drinking water sources.

**Supplementary Information:**

The online version contains supplementary material available at 10.1186/s12940-021-00755-6.

## Introduction

Cyanobacteria are widely distributed in the environment and are problematic in aquatic systems when they create dense assemblages (blooms) or produce potent secondary metabolites (cyanotoxins) that are harmful to animals, plants, and humans [1, 2]. Driven by climate and anthropogenic alterations, cyanobacterial blooms may change in frequency [3], extent [4], and magnitude [5] in many areas, which pose a threat to the sustainability of aquatic ecosystems as well as to human health [2, 6–8].

Humans can be exposed to cyanobacterial harmful algal blooms (cyanoHABs) via drinking water [6, 9–11]. CyanoHABs and cyanotoxins are found in source waters worldwide [6, 12–14], posing a global health risk. The most common reported acute health effect following cyanotoxin exposure through drinking water is gastrointestinal (GI) illness [15]. Recreational water exposure to cyanobacteria and cyanotoxins can result in multiple non-specific acute illnesses such as GI, respiratory, dermal, otic, neurological, musculoskeletal, and other signs and symptoms such as fever and anorexia [16, 17].

Measurement and ascertainment of cyanoHAB exposure is an important element during evaluation of the risk of cyanoHABs to human health. Standard exposure assessment may include in situ water sampling, cyanobacteria cell count, and quantifying toxin concentrations. These methods are often time-consuming, costly, and only feasible on a small geographic or temporal scale. Although satellite observations cannot detect toxins [18], they can assist in quantifying cyanobacteria abundance near the water surface. The use of remote sensing is a potentially attractive, low cost approach to characterizing the risk of human exposure to cyanoHABs [19]. Satellite remote sensing has been used to detect harmful algal blooms in large inland water bodies such as the Great Lakes in North America [20–22]. Recently, the use of remote sensing for cyanoHABs identification in smaller lakes using satellites has improved [23]. Satellite, sensor, and algorithm improvements now allow for the study of numerous inland lakes, improving our ability to remotely assess cyanoHABs in water bodies that serve as sources of drinking water or as recreational venues [3]. Satellite images acquired by the European Space Agency’s (ESA’s) MEdium Resolution Imaging Spectrometer (MERIS) can measure phytoplankton spectral signatures [24], and are now used to estimate cyanobacteria abundance in water [5, 25, 26].

The objective of this study is to explore the use of satellite-derived cyanobacteria abundance for exposure assessment and analysis of potential associations with human health effects. We used emergency room (ER) visit data to detect temporally- and spatially-associated acute illnesses in a human population served by a cyanoHAB-impacted drinking water source. We analyzed the three main classes of acute illnesses that could result from exposure to cyanobacterial blooms: GI, respiratory, and dermal illnesses [16, 27, 28].

## Methods

### Study area and water distribution system

**Fig. 1:**
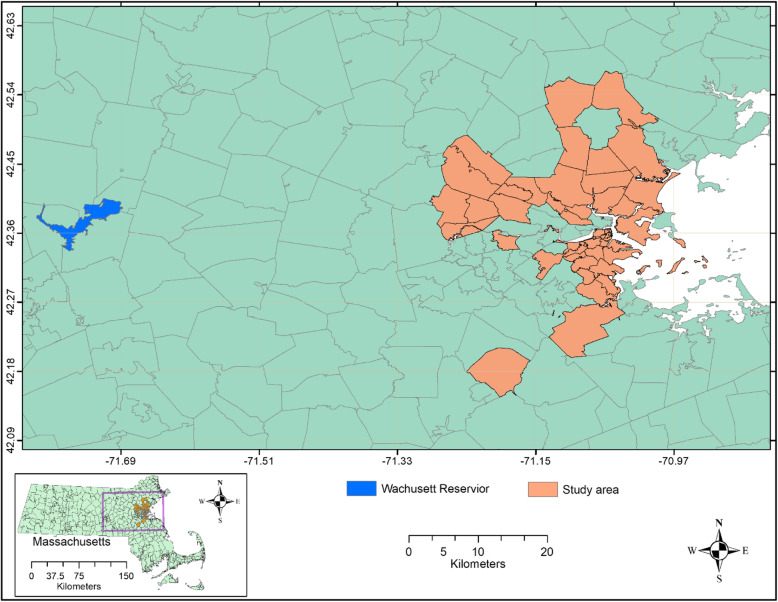
The study area in Massachusetts with boundaries of ZIP codes

Our study area included the city of Boston and other cities and towns in the Boston Metropolitan Area that receive municipal drinking water from the Massachusetts Water Resources Authority (MWRA) sourced from the Wachusett Reservoir located in Middlesex county, Massachusetts (Fig. 1). The Wachusett Reservoir is the second largest water body in the state. It serves as a source of drinking water for nearly 3 million people in the metropolitan Boston area (https://www.mass.gov/locations/wachusett-reservoir). The water is treated at the John J. Carroll Water Treatment Plant. The water is unfiltered; ozone is used as a primary disinfectant and chloramine as a secondary disinfectant (http://www.mwra.com/04water/html/watsys.htm). Twenty-two towns, located within 20–35 miles (32–56 km) of the reservoir intake, that also receive drinking water only from Wachusett Reservoir, were selected for inclusion in this study. The distance limits were used in order to ensure similar water residence times of approximately 2 days in the distribution system [29].

### Health data

Data on ER visits for GI, respiratory, and dermal illnesses during 2008–2011 were obtained from the Massachusetts Center for Health Information Analysis (CHIA) (http://www.chiamass.gov/), which accepts the submission of health data from approximately 70 Massachusetts hospitals. These anonymous, publicly-available, administrative health data were determined not to be personally identifiable human subjects data by the Human Subjects Research Protocol Officer for the U.S. Environmental Protection Agency (USEPA), and therefore are exempt from review by the USEPA’s Institutional Review Board of record. Administrative data included diagnostic code (International Classification of Diseases, Ninth Revision (ICD-9)), registration date, age, and the patients’ residential Zone Improvement Plan (ZIP) code. Patient residence in the study area determined their inclusion in the analysis. Eligible patients may have visited hospitals outside of the study area. We extracted the records on residents of the 22 towns included in the study using their ZIP codes. GI illnesses included ICD-9 codes 001–009.9, 558.9, 787.91, 787.01, and 787.03. Respiratory illnesses included ICD-9 codes 460, 461, 465.9, 493, 786.2, 786.05, and 786.07. Dermal illnesses included ICD-9 codes 782.1, 136.9, 686.9, 692, and 691.8 (see the description for these codes in Table S1). The response variable was the total daily number of patient visits for each category of illness in the study area.

### Cyanobacterial bloom data

Cyanobacteria concentration at the Wachusett Reservoir water intake were estimated using satellite images from MEdium Resolution Imaging Spectrometer (MERIS), an ESA sensor onboard the Envisat satellite which offers 300-m spatial resolution at nadir, where nadir is defined as the point on Earth’s surface directly below the satellite. The MERIS data archive includes a consistent time series over the study region for the years 2008–2011 with an image collected approximately every 3 days, and a usable image collected every 5 days on average when taking into account cloud, snow, and ice cover, which confound the satellite signal. Image processing was performed following the previous reported method [25, 30] and estimation of the cyanobacteria index (CI_cyano) followed Lunetta et al. 2015 [31]. Multiple inland water satellite algorithms could be considered to monitor cyanobacteria, however the updated CI_cyano from Lunetta et al. 2015 [31] was used here because it was validated across six New England states, including Wachusett Reservoir and several other lakes throughout Massachusetts. Additional validation efforts include agreement with state health advisories [32], state reported toxins and cell counts [33], and agreement against expected seasonality [25]. Only pixels within a 900-m buffer of the water intake that fell completely within the reservoir and excluding mixed land and water pixels along the shoreline were included in this analysis. CI_cyano was then converted into cyanobacteria concentration in cells/mL (cyan C) using the following equation: cyan C = CI*10^8^ following Lunetta et al. 2015 [31]. At a 300-m spatial resolution, five satellite pixels fell within a 900-m buffer of the drinking water intake. For each satellite image, cyanobacteria concentrations were calculated as the maximum cyan C value of these five pixels.

Estimated maximum cyanobacteria concentration data were classified into three categories defined using the sample distribution of cyan C values. Category 1 included observations with cyanobacteria concentrations <= 10,000 cells/mL, which is the assumed detection limit of the sensor; this level contained 50 % of the observations. Category 2 included those above the estimated detection limit but below the 3rd quartile of the distribution (cyanobacteria concentrations <= approximately 109,468 cells/mL); this category contained about 25 % of the observations. Category 3 included those above the 3rd quartile of the distribution up to the maximum satellite-derived cyanobacteria concentrations of 575,440 cells/mL and contained about 25 % of the observations.

### Covariates

Daily mean temperature and precipitation data for the Boston area were obtained from the PRISM Climate Group (http://www.prism.oregonstate.edu/explorer/). We selected one grid (4 km × 4 km, longitude: 42.3312, latitude: − 71.0761) to represent the weather conditions in the study area. Air pollution data were obtained from USEPA’s outdoor air quality website (https://www.epa.gov/outdoor-air-quality-data/download-daily-data). These data included daily concentrations of carbon monoxide (CO), nitrogen dioxide (NO_2_), sulfur dioxide (SO_2_), ozone (O_3_), and fine particulate matter (PM_2.5_) from a single air quality monitoring site (AQS) (ID: 250250042) in the study area. Given that the spatial variability of weather and air pollution data is relatively small, we assumed that these parameters were representative of the entire study area. ER visit counts differed from school vacation to school year and among days of the week and holidays [34, 35]. We created two binary variables: school days (all days in the school year including weekends) and holidays. In addition, we created dummy variables for each day of the week.

### Statistical analysis

We conducted exploratory data analyses, including plotting distributions of major variables, visually assessing temporal patterns, conducting correlation analysis of potential associations between temporal factors, such as day of the week, calendar month, holidays, meteorological variables and health data, and autocorrelation analysis of the health data. Time-series analysis of data was employed to assess associations between cyanobacteria concentration and lagged daily counts of illness as illness count data and meteorological data were daily while valid satellite images suitable for estimating cyanobacteria data were available at a lower frequency (on average, once per 5 days and often at irregular intervals). In order to fully use the available daily data in a time-series analysis, we conducted a regression analysis in two stages. First, we developed a predictive autoregressive generalized additive model (GAM) for each Poisson-distributed health outcome (respiratory, GI and dermal illness counts). All models included the spline function of time to account for seasonality and an autoregressive component (lagged daily counts of ER visits) at a 1-day lag. Other covariates tested included daily weather variables, air quality variables and indicator variables for weekdays, school days, and holidays as predictors. We used Akaike information criterion (AIC) to select the predictors for final stage one models [36].

Residuals from the first stage model were lacking temporal trends, seasonal patterns and serial autocorrelation. They were generally homoscedastic and normally distributed (Fig. 3). This suggests that the first stage analysis removed the effects of potential time-varying confounders, such as seasonality.

Next, residuals from the first stage models were regressed against original categorized cyanobacteria concentration data at lags from 0 to 6 days using univariable models for normally distributed (Gaussian) outcomes. As exploratory analysis of data demonstrated that cyanobacteria data for the winter period included many missing observations or values below the detection limit of the sensor, the second stage regression models excluded data for December, January and February. It has been shown previously that remotely sensed cyanobacterial bloom ascertainment is less available in winter due to spectral interference by ice or snow, particularly in northern latitude states such as Massachusetts [25].

## Results

**Fig. 2:**
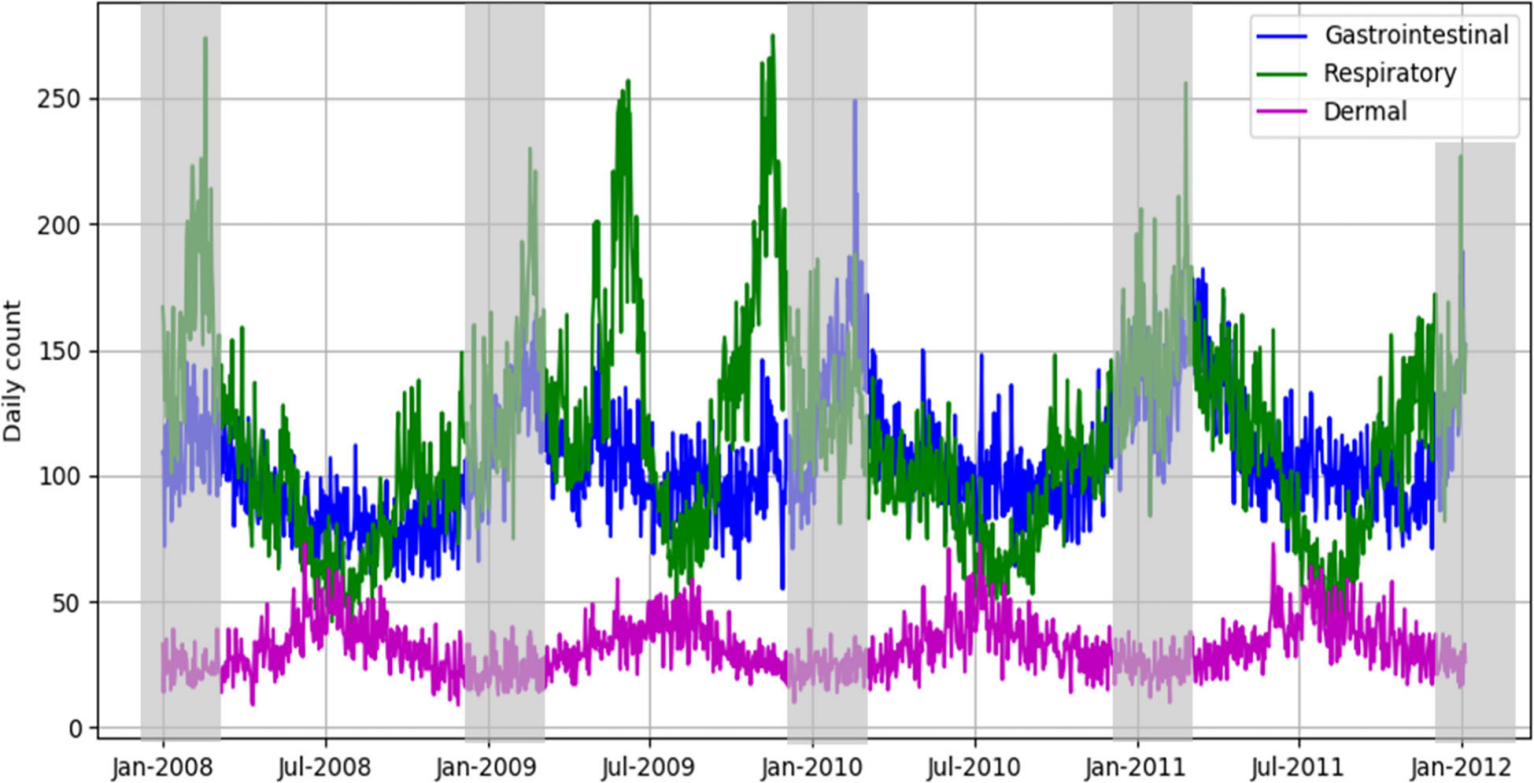
The daily counts of gastrointestinal, respiratory and dermal illnesses. The shadowed data in December, January and February were not used in the statistical analysis

The daily counts of GI, respiratory, and dermal illnesses during 2008–2011 are illustrated in Fig. 2. Visits for GI and respiratory illnesses included in this study generally peaked in the winter and their seasonal curves largely overlapped, although counts of respiratory illnesses had larger temporal variability. Counts of ER visits for GI illness averaged 72.6 (±14.9) per day, and count of respiratory illness visits averaged 79.1 (±30.0) per day (Table 1). In contrast, the daily count of dermal illness visits was much smaller, with a mean value of 24.2 (±7.4) per day; dermal illness visits were slightly higher in the summer compared to the winter.

**Table 1:** Descriptive statistics of variables used in the study (Data in December, January and February were excluded)

Variable	N	Mean	Std Dev	Minimum	Maximum
Daily count of gastrointestinal illness	1100	72.57	14.85	36	132
Daily count of respiratory illness	1100	79.13	29.88	27	206
Daily count of dermal illness	1100	24.24	7.38	6	55
Daily count of cyanobacteria cells (maximum from 5 pixels)	273	76,280	101,572	10,000	575,440
Daily O_3_ (ppm)	1100	0.03	0.01	0.005	0.07
Daily SO_2_ (ppb)	1100	3.63	3.71	0	35.9
Daily NO_2_ (ppm)	1100	29.23	9.85	8	68
Daily CO (ppm)	1100	0.36	0.18	0	1.9
Daily PM2.5 (μg/m^3^)	1100	9.48	5.30	1.1	57.4
Daily precipitation (mm)	1100	4.00	9.94	0	114.89
Daily mean temperature (°C)	1100	14.73	7.53	−8	30.6

**Fig. 3:**
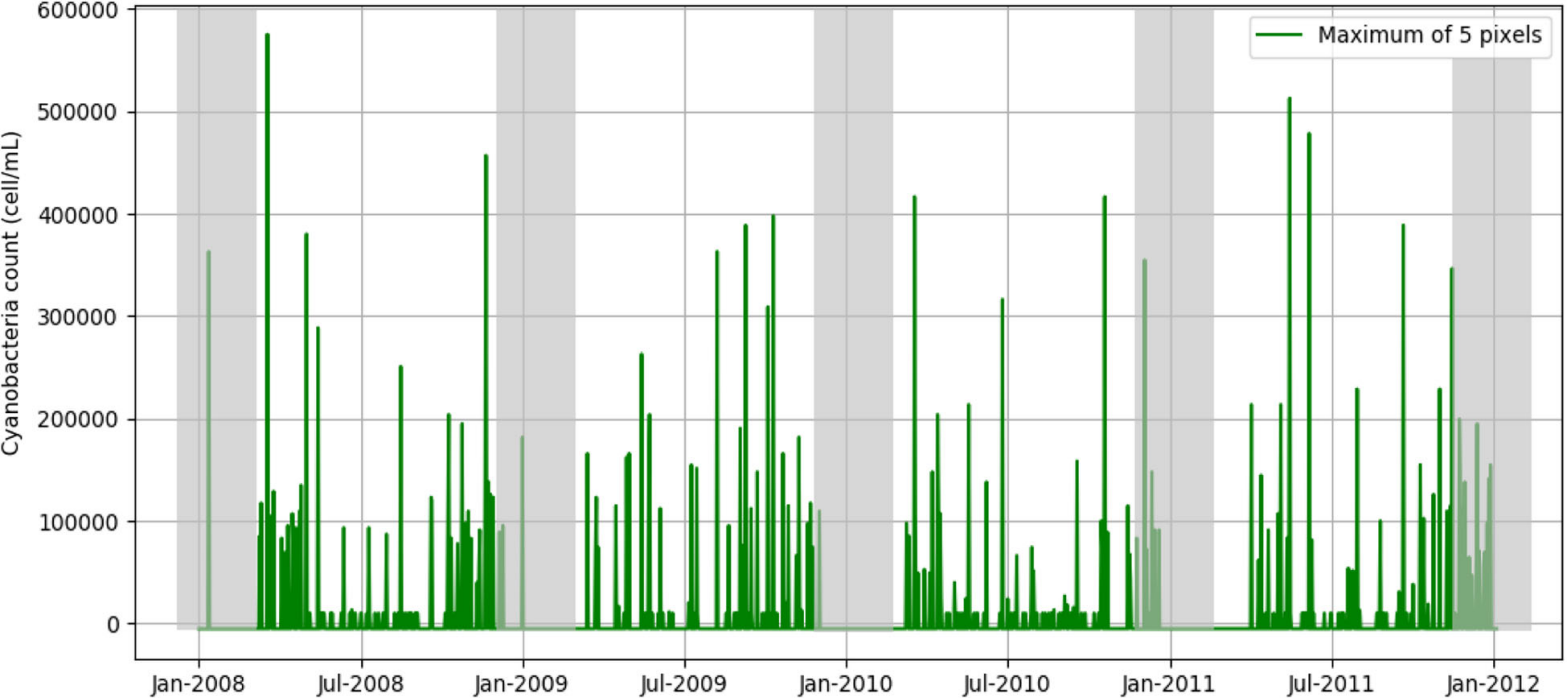
Estimates of cyanobacteria concentrations at the Wachusett drinking water intake during 2008–2011. Cyanobacteria concentrations were calculated as the maximum value of the five satellite pixels within a 900 m buffer of the drinking water intake. The shadowed data in December, January and February were not used in the statistical analysis

The estimated daily counts of remotely sensed cyanobacteria concentration near the drinking water intake during 2008–2011 are shown in Fig. 3. Shaded areas on this graph indicate winter months (December, January, and February) when the time-series of cyanobacteria concentrations contained many missing data due to unfavorable meteorological conditions (e.g., snow and ice cover). These data were excluded from stage 2 analysis.

Daily counts of GI illness had a significant positive correlation with school days, the concentrations of NO_2,_ and cyanobacteria concentration, but a significant negative correlation with O_3_, PM_2.5_, and daily mean temperature. Similarly, the daily count of respiratory illness visits had a significant positive correlation with school days, the concentrations of NO_2_, CO, and cyanobacteria concentration, but a significant negative correlation with O_3_, PM_2.5_, and daily mean temperature. Daily counts of dermal illness were negatively correlated with school days, SO_2_, NO_2_, CO, precipitation, and cyanobacteria concentration, but positively correlated with holidays, O_3_, PM_2.5_, and daily mean temperature (Table 2).

**Table 2:** Pearson correlation (r) between the counts of three types of illnesses and predictive variables (Data in December, January and February were excluded); p represents *p*-value and n represents the sample size. Bolded results represent significant correlations at the level of 0.001

Variables		Gastrointestinal Illness	Respiratory illness	Dermal illness
School days (yes/no)	r	**0.166**	**0.537**	**−0.461**
	p	**< 0.001**	**< 0.001**	**< 0.001**
	n	1100	1100	1100
Holiday (yes/no)	r	− 0.028	0.052	**0.087**
	p	0.348	0.087	**0.004**
	n	1100	1100	1100
O_3_	r	−0.056	**−0.218**	**0.357**
	p	0.066	**< 0.001**	**< 0.001**
	n	1100	1100	1100
SO_2_	r	0.030	**0.095**	**−0.135**
	p	0.320	**0.002**	**< 0.001**
	n	1100	1100	1100
NO_2_	r	**0.112**	0.045	**−0.158**
	p	**< 0.001**	0.129	**< 0.001**
	n	1100	1100	1100
CO	r	0.052	**0.219**	**−0.135**
	p	0.084	**< 0.001**	**< 0.001**
	n	1100	1100	1100
PM_2.5_	r	**−0.176**	**−0.182**	**0.264**
	p	**< 0.001**	**< 0.001**	**< 0.001**
	n	1100	1100	1100
Daily precipitation	r	0.001	−0.053	**−0.081**
	p	0.971	0.080	**0.007**
	n	1100	1100	1100
Daily mean temperature	r	**−0.214**	**−0.450**	**0.561**
	p	**< 0.001**	**< 0.001**	**< 0.001**
	n	1100	1100	1100
Cyanobacteria concentration (maximum from 5 pixels)	r	**0.150**	**0.152**	**−0.200**
	p	**0.013**	**0.012**	**0.001**
	n	273	273	273

**Fig. 4:**
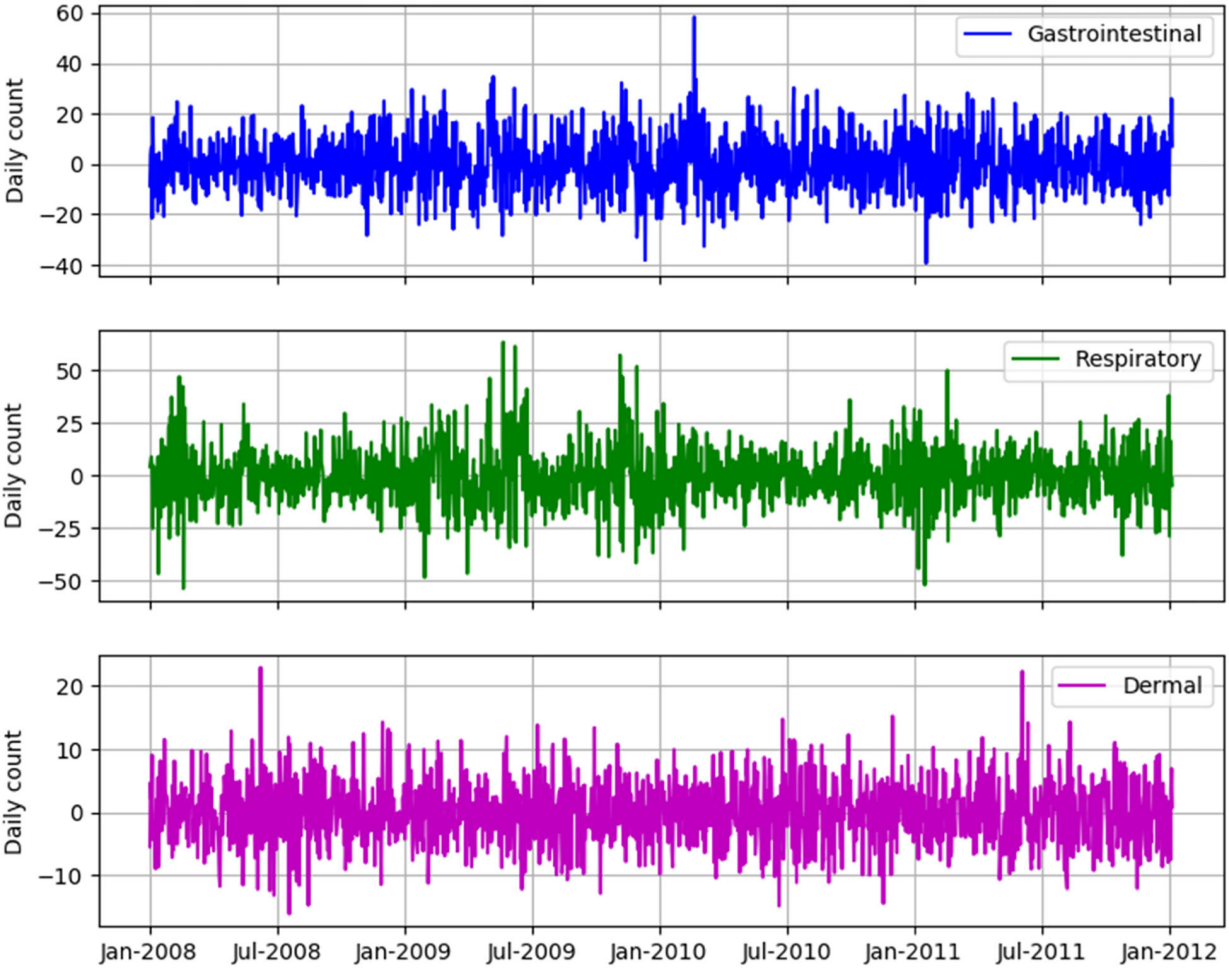
The residuals from the first stage model for emergency room visits: gastrointestinal, respiratory and dermal illnesses

The results from the first stage model for the three types of illnesses (GI, respiratory, dermal) are shown in Table 3. Days of the week and autoregression components, as well as spline functions of time, were significant predictors of daily counts of illness in all three models. Different meteorological and air pollution variables were retained in final stage 1 models for respiratory, dermal, and GI illnesses. The model residuals from the first stage models for the three types of illnesses are shown in Fig. 4. All residuals are approximately homoscedastic and normally distributed, lacking temporal trends and seasonal patterns.

**Table 3:** Results from the stage 1 model for emergency room visits for illnesses; p represents the *p*-value. Each model considered a constant sample size of 1460

Types of Illnesses	Variables	Mean Estimate	Standard Error	*P*
Gastrointestinal illness	Visit count on the previous day	0.003	0.000	< 0.001
	Monday	−0.022	0.010	0.032
	Tuesday	0.030	0.010	0.002
	Wednesday	0.115	0.010	< 0.001
	Thursday	0.047	0.010	< 0.001
	Friday	0.037	0.010	< 0.001
	O_3_	−0.619	0.314	0.049
	Average mean temperature	0.005	4.00*10^−4^	< 0.001
	Index of days	9.73*10^−5^	7.68*10^−6^	< 0.001
Respiratory illness	Visit count on the previous day	0.005	9.83*10^−6^	< 0.001
	Tuesday	−0.067	0.009	< 0.001
	Wednesday	−0.092	0.00932	< 0.001
	Thursday	−0.111	0.00916	< 0.001
	Friday	−0.099	0.00934	< 0.001
	Saturday	−0.030	0.00923	0.001
	School days	0.167	0.00855	< 0.001
	Holidays	−0.138	0.01624	< 0.001
	Precipitation	−0.001	3.00*10^−4^	0.004
	Index of days	−1.73*10^−6^	6.77*10^−6^	0.799
Dermal illness	Visit count on the previous day	0.002	0.001	0.010
	Tuesday	−0.094	0.017	< 0.001
	Wednesday	− 0.137	0.017	< 0.001
	Thursday	−0.178	0.017	< 0.001
	Friday	−0.189	0.018	< 0.001
	Holidays	−0.183	0.033	< 0.001
	O_3_	1.535	0.571	0.007
	NO_2_	0.002	0.001	0.006
	PM_2.5_	0.003	0.001	0.011
	Precipitation	−0.002	0.001	0.010
	Average mean temperature	0.008	0.001	< 0.001
	Index of days	8.10*10^−5^	1.73*10^−5^	< 0.001

Note: Holidays and each weekday (e.g., Monday, Tuesday, etc) are binary variables (Yes = 1, No = 0). For holidays, the value 1 was used as the reference, for each weekday, the value 0 was used as the reference

The results from the second stage models show that there were no significant associations between cyanobacteria concentrations and daily visits for GI illness at all time lags analyzed (Table 4). There was a significant positive association between satellite-derived cyanobacteria abundance and visits for respiratory illnesses at the two-day lag (Table 5). The adjusted effect associated with the highest category of cyanobacteria concentration was 4.3 additional cases with the 95% CI: 0.7–8.0 cases (*n* = 268). Associations at all other lags were not significant. Visits for dermal illnesses were not significantly associated with the level of cyanobacteria concentrations (Table 6). In addition, we divided the population of water consumers in different age groups (children, adults, and the elderly) and conducted stratified analysis using the same statistical models for each stratum. We did not find significant associations in individual groups.

**Table 4:** The association between cyanobacteria concentration (maximum estimate based on five pixels) and the cases of gastrointestinal illnesses (sample size = 268); p represents the *p*-value

Lag (day)	Category of cyanobacteria cells^a^	Regression coefficient	95% Confidence Interval		*P*
0	1	Reference			
	2	0.54	−2.23	3.31	0.703
	3	1.55	−1.45	4.55	0.312
1	1	Reference			
	2	−0.21	−3.01	2.59	0.883
	3	−2.24	−5.27	0.80	0.149
2	1	Reference			
	2	−0.29	−3.04	2.46	0.837
	3	−1.30	−4.27	1.67	0.392
3	1	Reference			
	2	−1.89	−4.68	0.90	0.185
	3	−0.44	−3.46	2.57	0.774
4	1	Reference			
	2	−0.44	−3.23	2.34	0.755
	3	−1.70	−4.73	1.33	0.271
5	1	Reference			
	2	−2.75	−5.63	0.13	0.062
	3	−1.58	−4.71	1.56	0.324

^a^Category 1: <=10,000 cell/mL; Category 2: above 10,000 cell/mL but below or equal to 109,468 cell/mL; and Category 3: > 109,468 cell/mL

**Table 5:** The association between cyanobacteria concentration (maximum estimate from five pixels) and the cases of respiratory illnesses (sample size = 268); p represents the *p*-value

Lag (day)	Category of cyanobacteria cells^a^	Regression coefficient	95% Confidence Interval		*P*
0	1	Reference			
	2	−2.66	−6.31	0.99	0.153
	3	−1.56	−5.51	2.39	0.440
1	1	Reference			
	2	−2.85	−6.25	0.54	0.100
	3	−2.79	−6.47	0.90	0.138
2	1	Reference			
	2	1.78	−1.62	5.18	0.304
	3	**4.32**	**0.65**	**7.99**	**0.021**
3	1	Reference			
	2	−0.55	−4.09	3.00	0.762
	3	0.27	−3.56	4.10	0.890
4	1	Reference			
	2	−1.69	−5.22	1.84	0.348
	3	−2.72	−6.55	1.12	0.166
5	1	Reference			
	2	−0.68	−4.48	3.12	0.727
	3	0.64	−3.50	4.77	0.763

^a^Category 1: <=10,000 cell/mL; Category 2: above 10,000 cell/mL but below or equal to 109,468 cell/mL; and Category 3: > 109,468 cell/mL

**Table 6:** The association between cyanobacteria concentration (maximum estimate from five pixels) and the cases of dermal illnesses (sample size = 268); p represents the p-value

Lag (day)	Category of cyanobacteria cells^a^	Regression coefficient	95% Confidence Interval		*P*
0	1	Reference			
	2	−0.73	−2.36	0.89	0.376
	3	−0.62	−2.38	1.14	0.493
1	1	Reference			
	2	−1.35	−2.74	0.05	0.059
	3	−0.90	−2.41	0.62	0.247
2	1	Reference			
	2	−0.09	−1.68	1.51	0.914
	3	−0.85	−2.57	0.88	0.337
3	1	Reference			
	2	−0.77	−2.36	0.82	0.344
	3	0.10	−1.62	1.81	0.913
4	1	Reference			
	2	−1.27	−2.69	0.14	0.078
	3	−0.30	−1.84	1.24	0.707
5	1	Reference			
	2	0.19	−1.28	1.67	0.799
	3	−0.59	−2.19	1.01	0.471

^a^Category 1: <=10,000 cell/mL; Category 2: above 10,000 cell/mL but below or equal to 109,468 cell/mL; and Category 3: > 109,468 cell/mL

## Discussion

This study evaluated acute illness among consumers of municipal drinking water associated with exposure to cyanobacterial blooms estimated using remotely sensed imagery. We investigated the association between satellite-derived cyanobacteria concentration in Wachusett Reservoir in Massachusetts during 2008–2011 and ER visits for three types of acute illnesses among residents of towns that receive drinking water from this reservoir.

To account for potential time-varying confounding factors, the two-stage modeling approach enabled utilization of daily data on health outcomes, weather, and air pollution. Regression analysis of detrended and normally distributed residuals from stage one autoregression GAM against cyanobacteria data demonstrated a lagged increase in the number of ER visits for respiratory illnesses associated with the highest category of cyanobacteria concentrations (> 109,468 cells/mL). It is important to highlight that the only significant association was detected at a 2-day lag corresponding to the average water residence time in the distribution system. This result suggests that drinking water from source water impacted by cyanobacterial blooms might be associated with respiratory symptoms following consumption. However, in contrast to other studies on effects associated with drinking water exposures to cyanobacteria, we did not observe significant associations between cyanobacteria in source water and ER visits for GI or dermal illnesses.

We are not aware of previously published reports of respiratory illness associated with exposure to cyanobacteria-impacted drinking water. However, several studies have examined the associations between respiratory illnesses and exposure to cyanobacteria in recreational water [16, 17, 27, 37–40]. Some of the previous reports describe severe respiratory illnesses such as pneumonia that resulted in hospital visits [27, 39, 40].

Hilborn et al. 2014 [16] reported that individuals recreating in or at cyanobacteria-impacted waters generally reported illness (including cough, wheeze, congestion and tight chest) on the day of exposure. In our study, the significant effect was observed at a two-day lag, which is the average time interval between water entering the intake at Wachusett Reservoir and water receipt by households in the study area. Fortunately, exposure to cyanobacteria via drinking water is not commonly reported because of the general effectiveness of drinking water treatment. If contamination does occur, there are multiple potential respiratory exposure pathways, including dishwashing, bathing, lawn watering, or other uses of tap water where a water aerosol is created [41].

Reports of GI and dermal illnesses associated with cyanobacterial blooms in drinking water have previously been published; however, findings from other studies were inconsistent. El Saadi et al. 1995 [42] found that the risk of GI symptoms was significantly increased for people drinking chlorinated water derived from a cyanobacteria-impacted river, and the risks of GI and dermal symptoms were increased for people using untreated water from the same river for bathing and dishwashing. However, the odds for developing GI and dermal symptoms was not significant for those exposed to recreational river water. Lévesque et al. 2014 [28] reported that exposure to cyanobacteria and microcystins in source water was linked to subsequent GI, muscle pain, skin, and ear symptoms, but symptoms were reported to be mild and did “not require a medical consultation.”

In this study, we did not observe a significant association between cyanobacteria and GI or dermal illnesses while some previous studies detected significant associations. A possible explanation for these discrepant results is that the exposure to cyanobacteria and health effects were defined in different ways in various studies. For example, Levesque et al. 2014 [28] counted cyanobacterial cells at multiple depths at sample sites in the drinking water source, and also measured cyanotoxin at those sampling sites. In their study, health effects among drinking water consumers were self-reported; therefore, they could capture mild symptoms which did not result in an ER visit. It is possible that any exposure to cyanobacteria in tap water in the Greater Boston area was associated with mild symptoms among water consumers which our study could not detect. This may also be because Levesque et al. 2014 [28] measured cyanotoxins in water while we used satellite data, which have two important limitations. First, satellite images cannot be used to measure toxins; and second, satellite images provide information for the surface layer of a waterbody. The satellite only measures the water’s surface, penetrating up to a depth of about 2 m in clear water [43] and less than 2 m in more turbid waters [9, 44]. This means that, depending on the depth of the drinking water intake, cyanobacteria concentrations at the water intake depth may not have been assessed in this study [3].

Beaudeau et al. 2014 [45] studied the same public water supply system, MWRA, but during a different time frame (1998–2008). They reported an association between the presence of cyanobacteria at the drinking water intake and hospital admissions for GI illness among the elderly 23–27 days later. Their study did not evaluate respiratory or dermal illnesses. Beaudeau et al. 2014 [45] also observed a decline in hospital admissions for GI illness after the water utility started treating drinking water with ozone in 2005 (prior to that, they used only chlorine for disinfection). Our study was wholly conducted after the introduction of ozone treatment. Therefore, it is possible that exposure to cyanobacteria via drinking water was diminished after 2005, as ozone is more effective at removing cyanobacterial toxins than chlorine [13].

Satellite remote sensing has a few additional limitations for human exposure assessment to cyanobacterial blooms. First, the availability and quality of satellite data is subject to the influence of cloud cover, snow and ice as well as other factors [3, 26]. Therefore, cyanobacterial bloom data were not available for every time the satellite passed over the study area. As our analysis was conducted in two stages, we were able to include full-year daily data on health, weather and air quality in stage one models. Thus, seasonality analysis was conducted using a full health dataset enabling us to properly characterize and remove seasonal patterns from health data. We had to exclude cyanobacteria data for December, January, and February from our stage two regression models because the presence of ice and snow or dense cloud cover could impact the accuracy of estimating cyanobacterial cell concentrations using satellite images. This limitation was due to the location of our study site in a northern part of the country where ice cover is formed on surface water bodies in winter.

Additionally, satellite-derived cyanobacteria concentration represents the maximum value within a 900-m buffer around the intake while actual cyanobacteria concentrations may be heterogenous spatially. Surface cyanobacteria concentrations may not accurately represent water quality at the actual drinking water intake depth.

This study has other inherent limitations. As an observational study, it could only demonstrate statistical associations but not causality. Although statistical analysis involved adjusting for multiple time-varying covariates and spline function of time, there could still be residuals from unmeasured time-varying factors correlated with cyanobacteria occurrence and daily disease counts. Furthermore, there could be ingestion exposure misclassification due to some individuals not drinking tap water. That might explain the lack of observed association between cyanobacteria and GI illness as such exposure misclassification would have biased the observed effect towards the null.

Despite the mentioned limitations, results from this study add to the existing literature of merging satellite technology for water quality with human health [46]. Our study has a few strengths. First, we simultaneously investigated three types of acute illnesses previously identified as being associated with cyanobacterial blooms, where previous studies have been largely restricted to the evaluation of gastrointestinal effects. A restricted a priori focus limits the ability to characterize health risks associated with exposure to drinking water impacted by cyanobacterial blooms. Second, we used daily health outcome data, which allowed us to characterize lagged effects of cyanobacterial blooms. Third, we analyzed associations between cyanobacteria concentration categories and illness counts at various lags from zero to 6 days and detected an association only at the two-day lag corresponding to the water residence time in the distribution system. Previous studies of recreational exposure demonstrated that illness symptoms occur on the day of exposure to cyanobacteria [16, 47]. We conducted statistical analysis at all 7 days in order to rule out confounding effects.

## Conclusions

We found a significant positive association between respiratory illnesses in the Greater Boston area and satellite-derived cyanobacteria concentration in source water at a time lag consistent with the water residence time in the distribution system. Because in situ monitoring data at the drinking water intake were not analyzed, this novel finding needs to be confirmed in future studies of health effects associated with exposure to cyanobacteria-impacted drinking water sources.

## Supplementary Information

**Additional file 1.**

## Data Availability

The health data used in this study are available from the Massachusetts Center for Health Information Analysis but restrictions apply to the availability of these data, which were used under data use agreement for the current study. The other data will be available from USEPA ScienceHub as the paper is published.

## Abbreviations

AIC: Akaike information criterion
AQS: Air quality monitoring site
CO: Carbon monoxide
ER: Emergency room
cyanoHABs: Cyanobacterial harmful algal blooms
GAM: Generalized additive model
GI: Gastrointestinal
ICD-9: International Classification of Diseases, Ninth Revision
MERIS: MEdium Resolution Imaging Spectrometer
MWRA: Massachusetts Water Resources Authority
NO_2_: Nitrogen dioxide
O_3_: Ozone
PM_2.5_: Fine particulate matter
SO_2_: Sulfur dioxide
USEPA: U.S. Environmental Protection Agency
