# Supplementary material for: Acute health effects associated with satellite-determined cyanobacterial blooms in a drinking water source in Massachusetts
Source: Environ Health. 2021 Jul 16;20:83. doi: 10.1186/s12940-021-00755-6 (PMC8285816; doi:10.1186/s12940-021-00755-6)
Supplement: Supplementary file 1 — Additional file 1. [file 12940_2021_755_MOESM1_ESM.docx]

**Acute health effects associated with satellite-determined cyanobacterial blooms in a drinking water source in Massachusetts**

Jianyong Wu^1*^, Elizabeth D. Hilborn^2*^, Blake A. Schaeffer^2^, Erin Urquhart^3^, Megan M. Coffer^1,4^, Cynthia J. Lin^1,5^, and Andrey I. Egorov^2^

1. Oak Ridge Institute for Science and Education, US EPA, Office of Research and Development, Research Triangle Park, Durham 27711, NC, USA
2. US Environmental Protection Agency, Office of Research and Development, Research Triangle Park, Durham 27711, NC, USA
3. Science Systems and Applications, Inc., NASA Goddard Space Flight Center, Greenbelt, MD, USA
4. Center for Geospatial Analytics, North Carolina State University, Raleigh, NC, USA
5. ICF International, Durham, NC 27713

Table S1. The description of ICD-9 codes for respiratory diseases interested in this study

| Type of diseases | ICD-9 codes | Description |
| --- | --- | --- |
| GI illness | 001-009.9 | Intestinal infectious diseases |
|  | 558.9 | Other and unspecified noninfectious gastroenteritis and colitis |
|  | 787.01 | Nausea with vomiting |
|  | 787.03 | Vomiting alone |
|  | 787.91 | Diarrhea |
| Respiratory illness | 460 | Acute nasopharyngitis (common cold) |
|  | 461 | Acute sinusitis |
|  | 465.9 | Acute upper respiratory infections of unspecified site |
|  | 493 | Asthma |
|  | 786.2 | Cough |
|  | 786.05 | Chest pain |
|  | 786.07 | Abnormal chest sounds |
| Dermal illness | 136.9 | Unspecified infectious and parasitic diseases |
|  | 686.9 | Unspecified local infection of skin and subcutaneous tissue |
|  | 691.8 | Other atopic dermatitis and related conditions |
|  | 692 | Contact dermatitis and other eczema |
|  | 782.1 | Rash and other nonspecific skin eruption |
